# Comparative Effectiveness of Fluid Resuscitation Strategies for Preventing Acute Kidney Injury in Critically Ill Patients: A Meta-Analysis

**DOI:** 10.7759/cureus.90060

**Published:** 2025-08-14

**Authors:** Ihtesham Shafiq, Margaret C Hastings, Hieu Q Vo, Syed M Raza, Syed F Zabiullah, Qazi Kamran Amin

**Affiliations:** 1 Department of Medicine, Division of Med Nephrology, The University of Tennessee Health Science Center, Memphis, USA; 2 Department of Internal Medicine, Rehman Medical Institute, Peshawar, PAK

**Keywords:** acute kidney injury, aki, fluid overload, fluid resuscitation, icu patients

## Abstract

Acute kidney injury (AKI) is a common complication in critically ill patients and is associated with high morbidity and mortality. This meta-analysis compares the effectiveness of different fluid resuscitation strategies in preventing AKI in critically ill patients. A systematic search was conducted in PubMed, MEDLINE, EMBASE, and the Cochrane database for randomized controlled trials comparing fluid resuscitation strategies in critically ill patients with AKI as an outcome. A random-effects meta-analysis was performed to calculate pooled ORs and risk ratios (RRs) with 95% CIs. Eight studies with 1,390 patients were included. There was no significant difference in AKI incidence between early goal-directed therapy (EGDT) and usual care (OR 0.90, 95% CI 0.71-1.13) or between crystalloids and colloids (OR 1.03, 95% CI 0.89-1.18). Mortality rates were similar between EGDT and usual care (RR 1.02, 95% CI 0.78-1.38) and between crystalloids and colloids (RR 1.03, 95% CI 0.93-1.14). The need for RRT did not differ significantly between strategies. Length of ICU stay was longer with EGDT than with usual care (MD 2.81 days, 95% CI 0.21-5.41), but was similar between crystalloids and colloids. This meta-analysis found no significant differences in AKI incidence, mortality, or need for RRT between fluid resuscitation strategies in critically ill patients. EGDT was associated with longer ICU stays compared to usual care. Further research is needed to optimize fluid management in critical care settings.

## Introduction and background

Acute kidney injury (AKI) is a common clinical complication in critically ill patients, particularly those admitted to the ICU or undergoing surgery, and is associated with prolonged hospital stays as well as high rates of morbidity and mortality [1]. AKI is reported in approximately two-thirds of ICU admissions [2]. Two common etiologies are shock and sepsis, which increase the risk of AKI by up to 80% [3].

According to the Kidney Disease: Improving Global Outcomes (KDIGO) criteria, which combine the RIFLE and AKIN classifications, AKI is defined as an increase in serum creatinine (SCr) of ≥0.3 mg/dL (≥26.5 μmol/L) within 48 hours, an increase in SCr to ≥1.5 times baseline within seven days, or urine output <0.5 mL/kg/h for six hours. The AKIN criteria are used to grade severity; for grade 3, the creatinine-based change in the definition must be met before SCr >4.0 mg/dL (>354 μmol/L) is considered [4]. AKI is typically diagnosed by decreased urine output and increased SCr levels [5]. Its occurrence, regardless of severity or clinical course, significantly reduces both short- and long-term survival [6], emphasizing the importance of preventive strategies in critically ill patients [7].

Prevention strategies for AKI generally follow multimodal clinical algorithms aimed at optimizing perfusion pressure and volume status to improve renal blood flow [5,8]. Low mean arterial pressure and high central venous pressure are not reliable indicators of fluid responsiveness, and results should be interpreted in the context of dynamic assessments and the broader clinical picture [9]. The development of protocolized hemodynamic resuscitation has been driven by uncertainty regarding fluid resuscitation endpoints. Notably, more conservative intravenous fluid administration strategies have been associated with a higher incidence of AKI [10]. Thus, fluid resuscitation management is crucial for maintaining hemodynamic stability and preserving renal function [6,11,12].

Fluid resuscitation strategies are employed in conditions such as trauma, postoperative recovery, and septic shock. Commonly used fluids include crystalloids (e.g., lactated Ringer’s solution and normal saline) and colloids (e.g., albumin and hydroxyethyl starch (HES)). Recent studies suggest that fluid type may influence AKI risk and exacerbate preexisting renal impairment [13]. For example, increased colloid use may raise AKI risk in critically ill patients with renal comorbidities [11,14,15]. Hartog et al. [16] reported that while synthetic colloids can be hazardous depending on cumulative dose, they may offer benefits in certain severely ill adults and children. Conversely, other studies recommend crystalloids over colloids, citing improved renal outcomes. Lewis et al. [17] found that crystalloids reduced the risk of AKI, mortality, and the need for renal replacement therapy (RRT) compared with colloids. However, excessive crystalloid administration can cause fluid overload and subsequent renal failure.

This complexity underscores the need for a comprehensive evaluation of the available evidence on fluid resuscitation strategies. Currently, there is limited comparative research on the relative efficacy of these strategies for AKI prevention. Therefore, this meta-analysis aims to evaluate the effectiveness of various fluid resuscitation strategies in preventing AKI among critically ill patients.

## Review

Methods

Protocol

This meta-analysis was conducted in accordance with the Cochrane Handbook for Systematic Reviews of Interventions [18] and the Preferred Reporting Items for Systematic reviews and Meta-Analyses (PRISMA) statement for screening and selecting research articles [19]. As only completed randomized controlled trials (RCTs) and observational studies were used, ethical approval was not required.

Data Sources and Search Strategy

Research papers relevant to the study aim were retrieved from PubMed, MEDLINE, EMBASE, and the Cochrane Library. The search used the MeSH terms “(effectiveness OR comparative effectiveness OR impacts OR outcomes) AND (fluid resuscitation strategies OR fluid management) AND (AKI OR acute kidney injury OR preventing AKI) AND (ICU admitted patients OR critically ill patients),” with English-language restrictions. The search period was January 2000 to October 2024. Reference lists of relevant review articles and retrieved trials were also manually screened.

Eligibility Criteria

Predefined selection criteria guided the screening process. Inclusion criteria were (1) studies involving critically ill patients in hospital or at home; (2) studies comparing groups receiving different fluid resuscitation or management strategies; (3) studies reporting prevention of AKI as a primary outcome; (4) RCTs or observational studies; and (5) studies published in English with full text available. Exclusion criteria were (1) studies involving patients with mild illness; (2) studies examining fluid overload or other therapies such as RRT without a comparison group; (3) studies reporting outcomes other than AKI prevention; (4) systematic reviews, meta-analyses, scoping reviews, or case studies; and (5) studies published in languages other than English or without full-text access.

Study Selection

All articles obtained through the database search were screened according to PRISMA guidelines. Two authors independently reviewed the titles and abstracts, excluding irrelevant articles. Full texts of potentially eligible studies were then assessed against the eligibility criteria, and those meeting the requirements were included in the pooled analysis.

Data Extraction

Two authors independently extracted data from included studies using a pre-piloted table. Extracted information included baseline characteristics (author, year, country, study population with mean age, study groups, follow-up period, fluid resuscitation type, and study design) as well as primary outcomes (incidence and severity of AKI, mortality rates, need for RRT, and length of hospital stay). For AKI, the definition used was an increase in SCr of at least twofold, or an SCr level ≥3.96 mg/dL with an increase of ≥0.5 mg/dL.

Quality Assessment

The risk of bias in the included studies was assessed using the Cochrane Library tool for RCTs [20]. Each study was evaluated across seven domains: (a) selection bias or random sequence generation; (b) allocation concealment; (c) performance bias or blinding of participants and personnel; (d) detection bias or blinding of the outcome assessor; (e) selective bias or selective reporting; and (f) other biases. Each domain was graded as low risk, high risk, or unclear. In addition, the Risk Of Bias In Non-randomized Studies of Interventions (ROBINS-I) tool was used to assess the risk of bias in non-randomized clinical studies [21]. ROBINS-I evaluates seven domains: selection of the control group, recruitment of participants, classification of interventions, deviations from intended interventions, missing data, measurement of outcomes, and selection of the reported result. The risk of bias was classified into five levels: low risk, moderate risk, serious risk, critical risk, and no information.

Data Analysis

Statistical analysis was conducted using Review Manager (RevMan, version 5.4; The Cochrane Collaboration). A random-effects model was applied due to significant heterogeneity among the included studies (I² > 50%). Study outcomes were reported as risk ratios (RRs), ORs, or mean differences, each with a 95% CI. The chi-square test was considered statistically significant when p < 0.1. Funnel plots were generated to assess publication bias [22].

Results

Search Results

**Figure 1:**
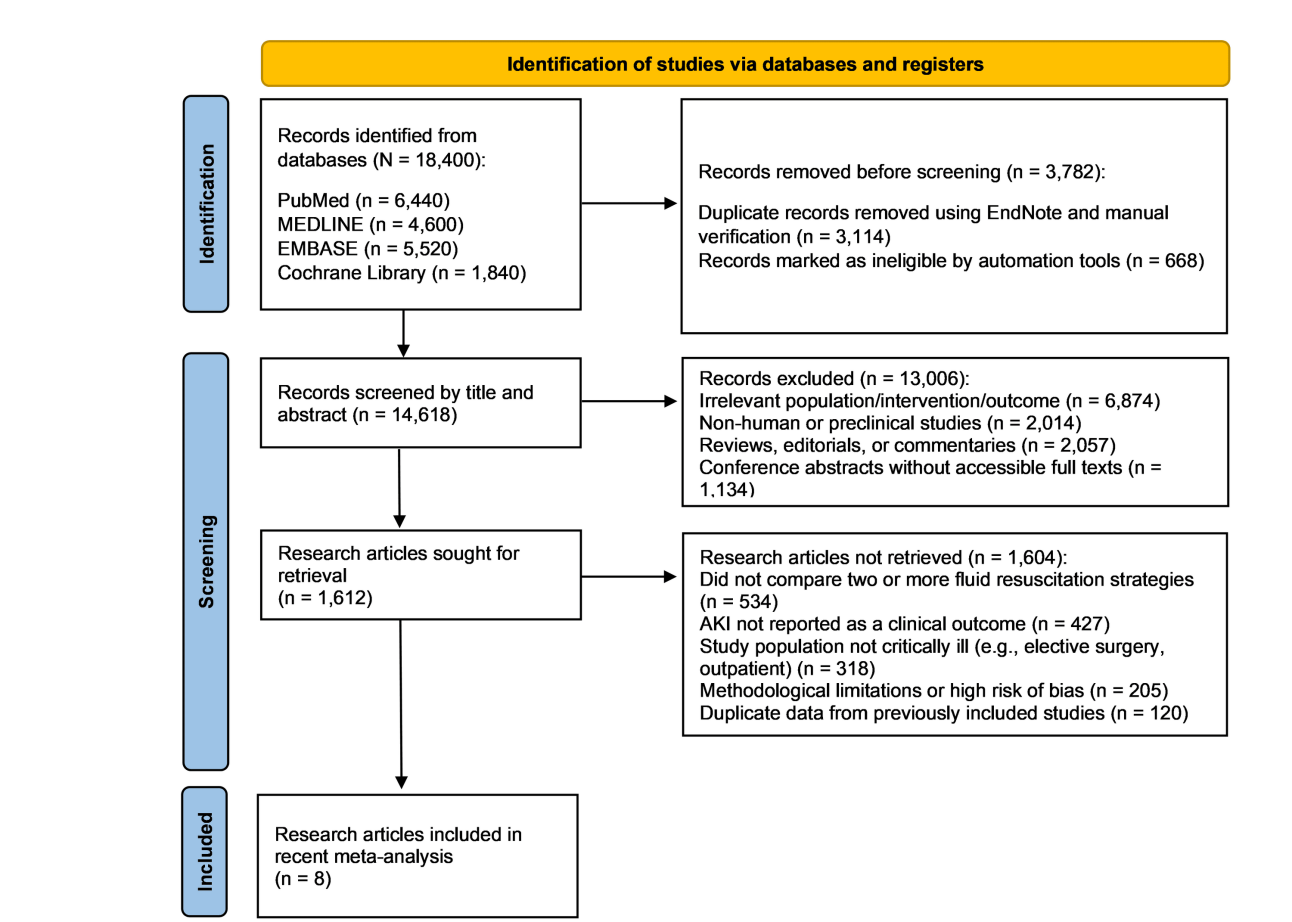
PRISMA 2020 flow diagram showing the screening and selection of included studies from database and register searches PRISMA: Preferred Reporting Items for Systematic reviews and Meta-Analyses

Research articles were identified and screened in accordance with the study aims, following PRISMA guidelines. A total of 18,400 research articles were retrieved from the databases mentioned above using MeSH keywords. After excluding 1,022 articles, 5,608 papers remained for screening. Of these, 1,055 articles were assessed for eligibility. Ultimately, eight studies met the inclusion criteria and were included in the analysis, as illustrated in Figure 1.

Characteristics of Included Studies

This meta-analysis included eight RCTs involving a total of 1,390 critically ill patients to compare the effectiveness of different fluid resuscitation strategies in preventing AKI. The main characteristics of the included studies are presented in Table 1. All studies were published between 2000 and 2024. Sample sizes varied considerably, ranging from 94 to 6,997 ICU-admitted patients, reflecting differences in study scope and recruitment feasibility in critically ill populations. The mean age of patients ranged from 18 to 60 years. Follow-up durations also differed, ranging from 48 hours to 90 days.

**Table 1:** Characteristics of included studies AKI: acute kidney injury; EGDT: early goal-directed therapy; NR: not reported; P: placebo; PiCCO: pulse-indicated continuous cardiac output; RCT: randomized controlled trial; RRT: renal replacement therapy; T: treatment

Author and year	Country	Study population with a mean age	Study design	Study follow-up	Types of fluid resuscitation	Incidence of AKI	Mortality	Need for RRT	Length of ICU stay
Young et al. (2015) [23]	New Zealand	2,278 patients: 1,152 received buffered crystalloid, 1,110 received saline	Randomized, double-crossover trial	90 days	Buffered crystalloid vs. saline	T: 102, P: 94	T: 87, P: 95	T: 38, P: 38	NR
Annane et al. (2013) [24]	France	2,857 ICU patients (60 years old): 1,443 received crystalloids, 1,414 received colloids	Pragmatic randomized trial	90 days	Crystalloids vs. colloids	NR	T: 493, P: 434	T: 181, P: 156	T: 1.8, P: 2.1
Wang et al. (2020) [25]	China	94 patients: 51 in the EGDT group, 43 in the PiCCO group	RCT	48 hours	EGDT vs. PiCCO	NR	T: 8, P: 6	T: 9, P: 5	T: 14.50 ± 5.78, P: 10.35 ± 3.50
Vaara et al. (2021) [26]	Finland	100 AKI patients: 49 in restrictive fluid management, 51 in usual care	Randomized controlled feasibility trial	72 hours	Restrictive fluid management vs. usual care	T: 29, P: 39	T: 9, P: 13	T: 6, P: 15	T: 13 days, P: 11.5 days
Finfer et al. (2004) [27]	New Zealand	6,997 patients: 3,497 received albumin, 3,500 received saline	Randomized, double-blind trial	28 days	Albumin vs. saline	T: 335, P: 329	T: 726, P: 729	NR	T: 6.5 ± 6.6, P: 6.2 ± 6.2
Silversides et al. (2022) [28]	United Kingdom	180 patients: 89 in fluid management, 90 in usual care	Randomized clinical feasibility trial	48 hours	Conservative fluid management vs. usual care	NR	T: 19, P: 14	NR	NR
Hjortrup et al. (2016) [29]	Denmark	151 ICU patients: 75 in fluid resuscitation, 76 in standard care	Randomized clinical feasibility trial	90 days	Restrictive fluid management vs. standard care	T: 27, P: 39	T: 25, P: 31	NR	NR
Kellum et al. (2015) [30]	USA	1,243 septic shock patients: 439 in EGDT, 456 in usual care	Randomized controlled trial	60 days	EGDT vs. usual care	T: 203, P: 206	T: 97, P: 94	T: 25, P: 18	NR

Across the included RCTs, the interventions compared were crystalloids versus colloids, crystalloids versus saline, early goal-directed therapy (EGDT) versus pulse-indicated continuous cardiac output, and restrictive fluid resuscitation versus usual care, in line with the study’s objectives. The studies were conducted in various countries, contributing to heterogeneity in patient populations, healthcare settings, and treatment protocols (Table 1).

Risk Bias Assessment

Of the eight included studies, four were assessed as low risk [26,27,29,30], and four as moderate risk [23-25,28]. No high-risk studies were identified, as shown in Appendix A and Appendix B.

Primary Outcomes

The primary outcomes of this meta-analysis were the incidence of AKI, mortality rates, length of ICU stay, and the need for RRT.

Incidence of AKI

**Figure 2:**
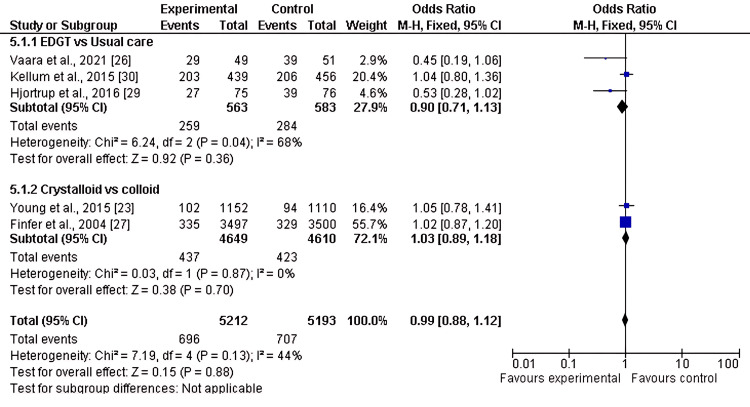
Forest plot of the incidence of AKI in two subgroups of fluid resuscitation strategies AKI: acute kidney injury

**Figure 3:**
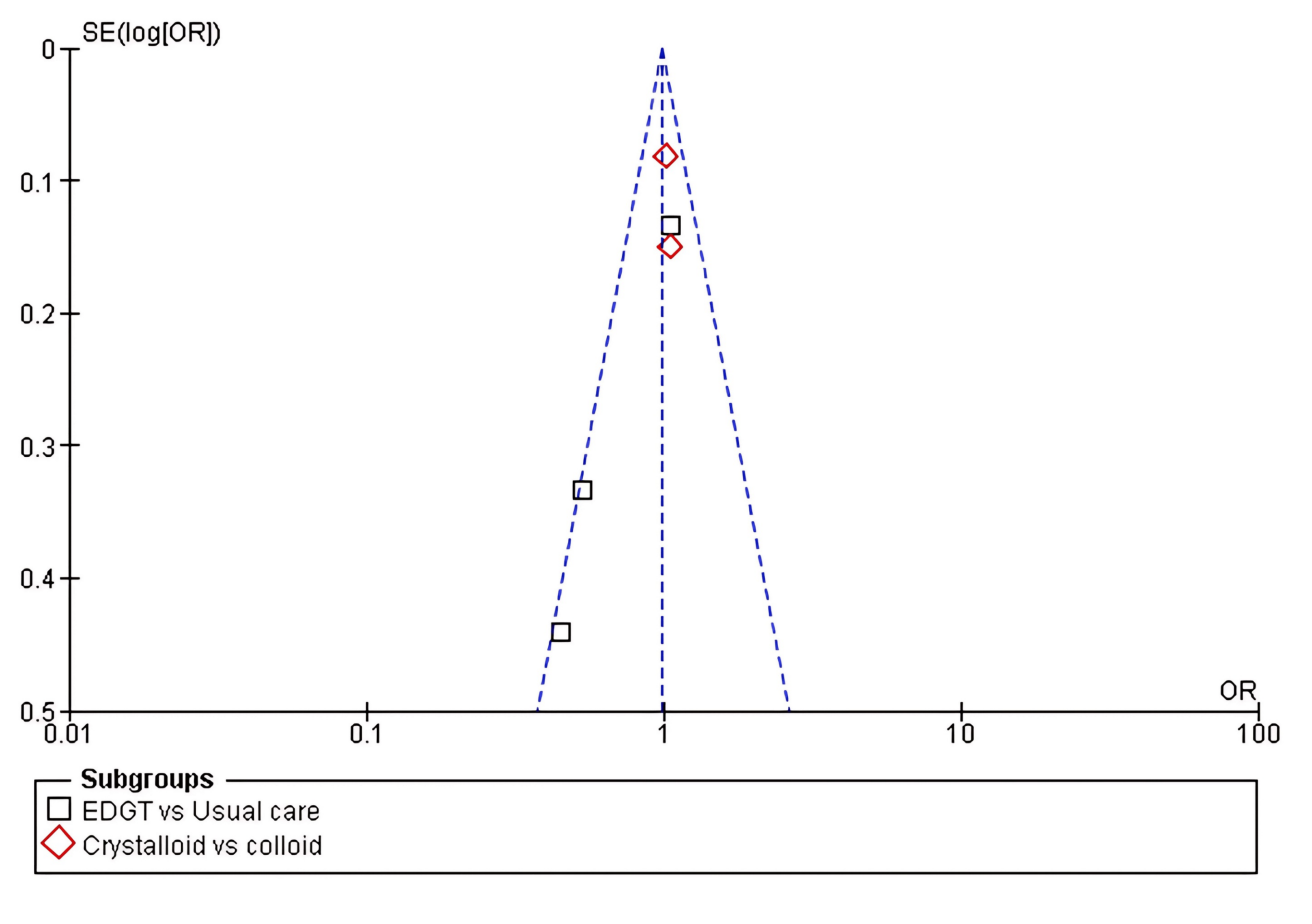
Funnel plot assessing publication bias across included studies The x-axis represents the effect size (OR), and the y-axis shows the standard error of the log OR. Subgroups include EGDT vs. usual care and crystalloids vs. colloids. SE(log(OR)): standard error of log OR

Five of the eight included studies reported the incidence of AKI as an outcome when comparing two or more fluid resuscitation strategies. The pooled analysis showed no significant difference in AKI incidence in the first subgroup, EGDT versus usual care (OR 0.90, 95% CI 0.71-1.13; p = 0.36). Similarly, no significant difference was observed in the second subgroup, crystalloids versus colloids (OR 1.03, 95% CI 0.89-1.18; p = 0.70), with moderate heterogeneity (I² = 44%), as shown in Figure 2 and Figure 3.

Mortality Rates

**Figure 4:**
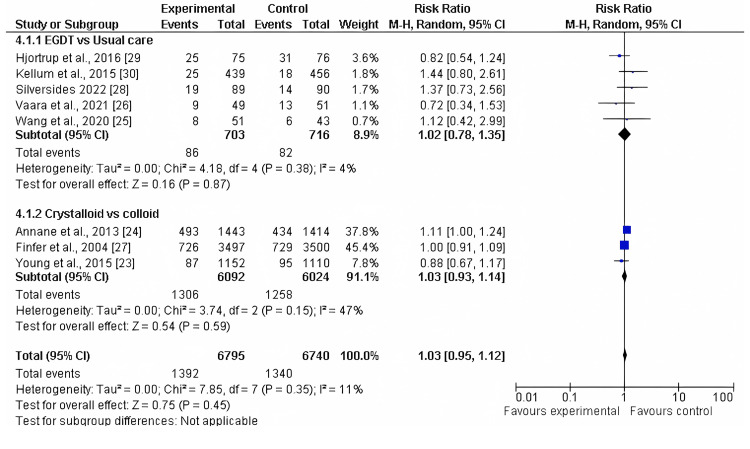
Forest plot of mortality rates in two subgroups of fluid resuscitation strategies

**Figure 5:**
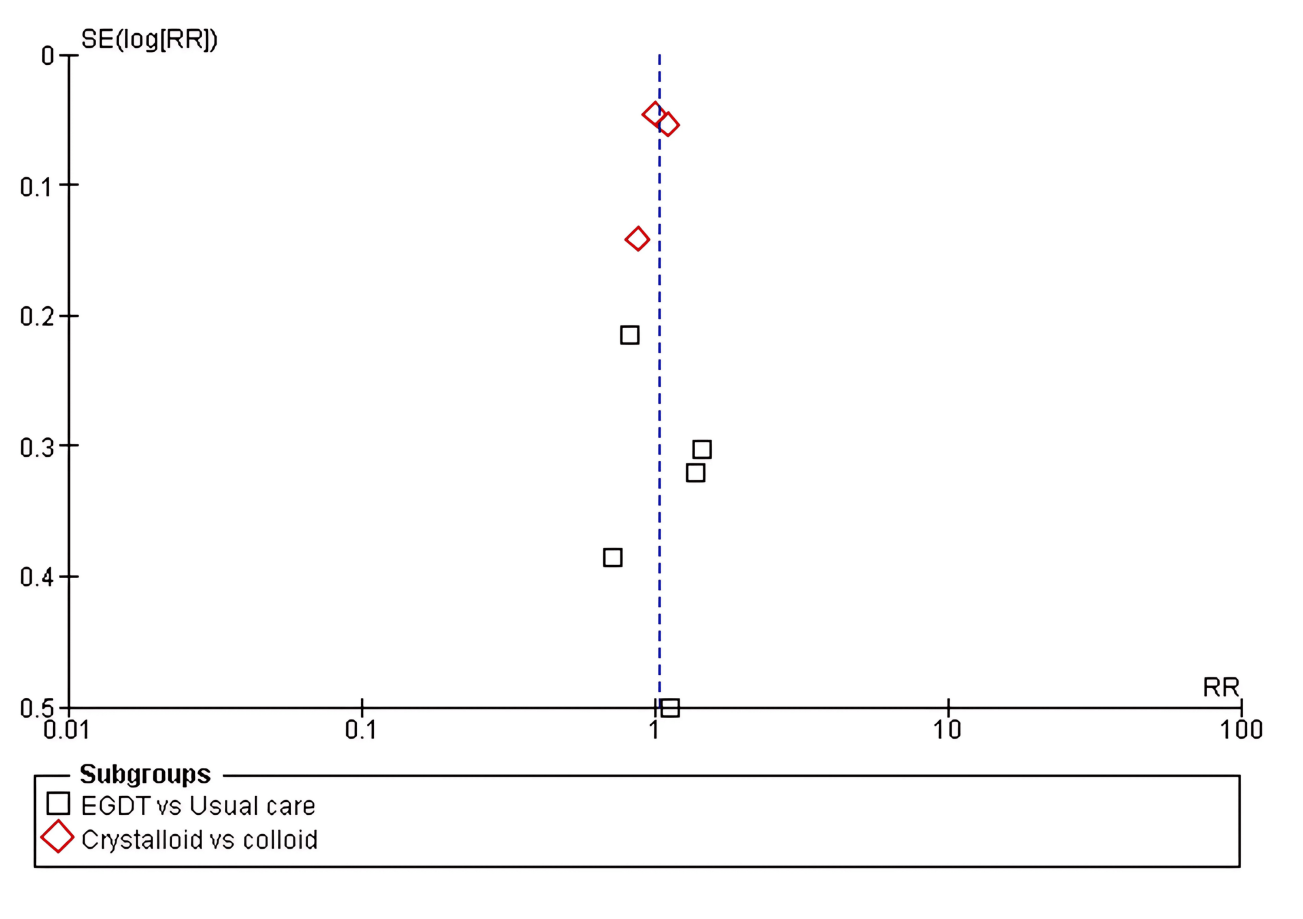
Funnel plot assessing publication bias across included studies The x-axis represents the effect size (RR), and the y-axis shows the standard error of the log-transformed RR. Each symbol represents a study. Subgroups include EGDT vs. usual care and crystalloids vs. colloids. EGDT: early goal-directed therapy; RR: risk ratio; SE(log(RR)): standard error of log risk ratio

All eight included studies reported mortality rates as an outcome when comparing two or more fluid resuscitation strategies. The pooled analysis showed no significant difference in the risk of mortality in the first subgroup, EGDT versus usual care (RR 1.02, 95% CI 0.78-1.38; p = 0.38). Likewise, no significant difference was found in the second subgroup, crystalloids versus colloids (RR 1.03, 95% CI 0.93-1.14; p = 0.35), with low heterogeneity (I² = 4%), as shown in Figure 4 and Figure 5.

Need for RRT

**Figure 6:**
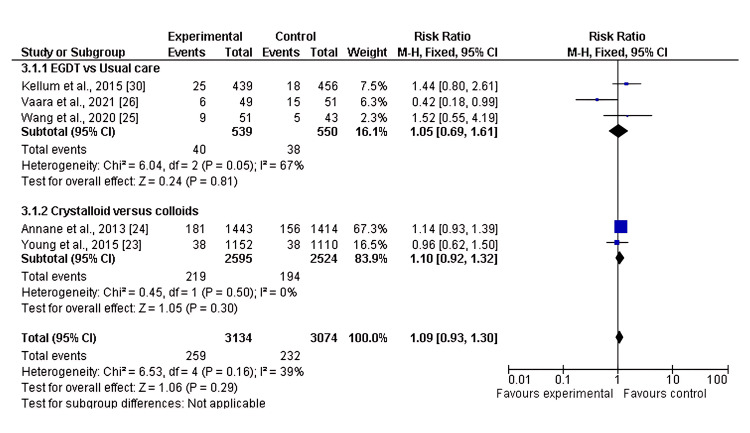
Forest plot of the risk of RRT in two subgroups of fluid resuscitation strategies RRT: renal replacement therapy

**Figure 7:**
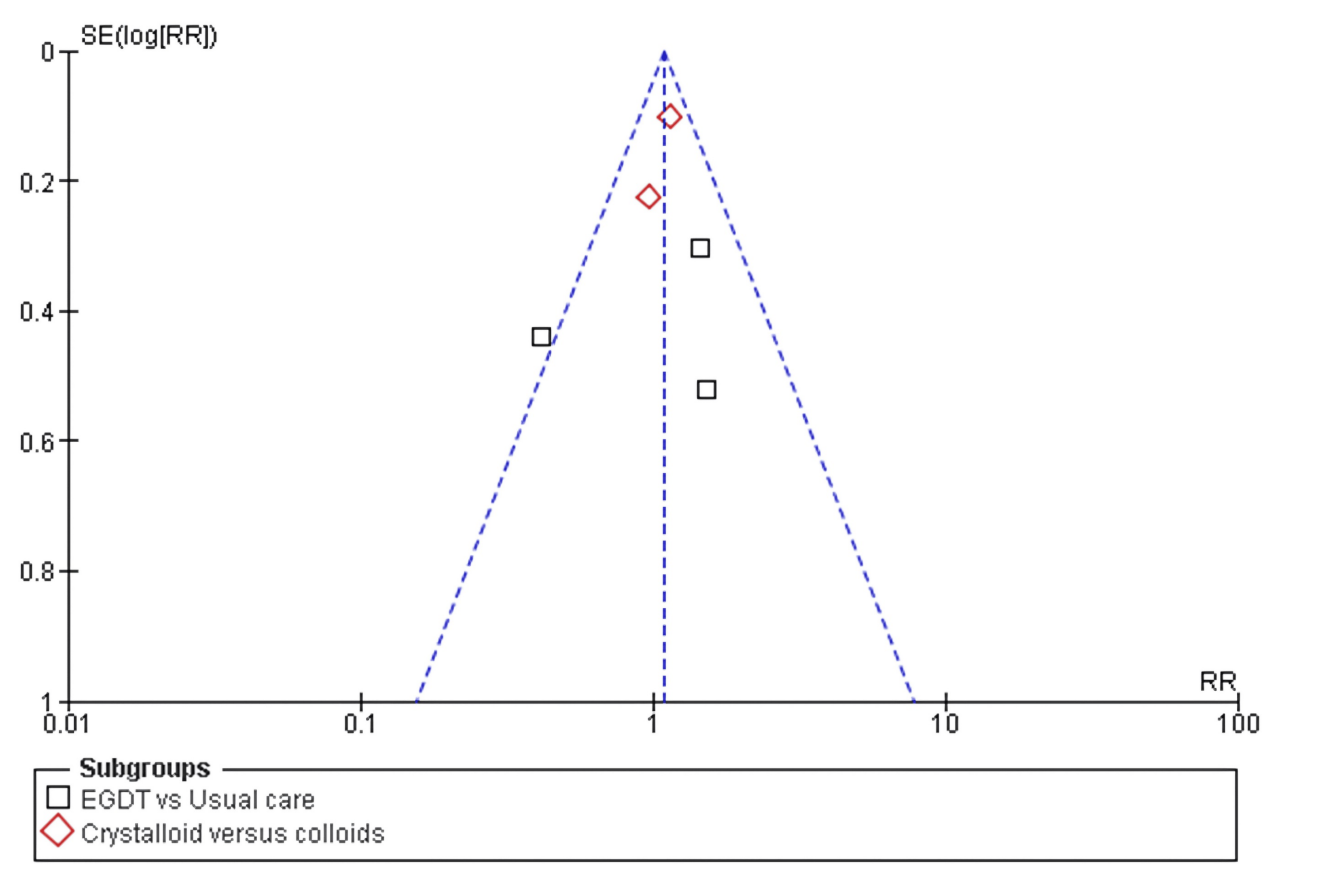
Funnel plot showing the distribution of studies comparing EGDT vs. usual care (black squares) and crystalloids vs. colloids (red diamonds) The plot suggests minimal publication bias, though interpretation is limited by the small number of studies. EGDT: early goal-directed therapy; RR: risk ratio; SE(log(RR)): standard error of log risk ratio

Of the eight included studies, five reported the need for RRT as an outcome when comparing two or more fluid resuscitation strategies. The pooled analysis showed no significant difference in the risk of RRT in the first subgroup, EGDT versus usual care (RR 1.05, 95% CI 0.69-1.61; p = 0.81). Similarly, no significant difference was observed in the second subgroup, crystalloids versus colloids (RR 1.10, 95% CI 0.93-1.30; p = 0.30), with moderate heterogeneity (I² = 39%), as shown in Figure 6 and Figure 7.

Length of ICU Stay

**Figure 8:**
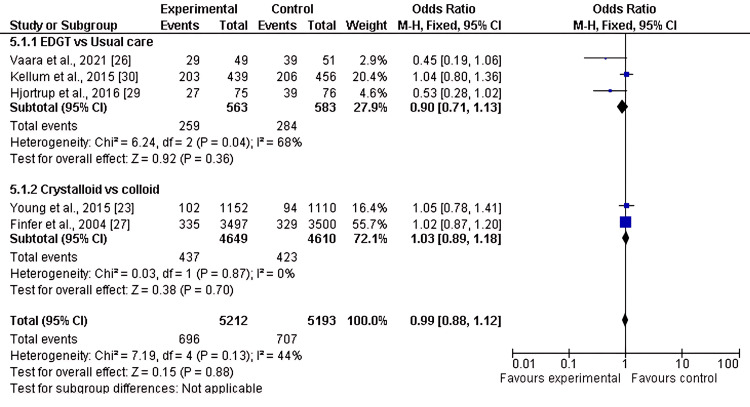
Forest plot of the mean difference in length of ICU stay in two subgroups of fluid resuscitation strategies

**Figure 9:**
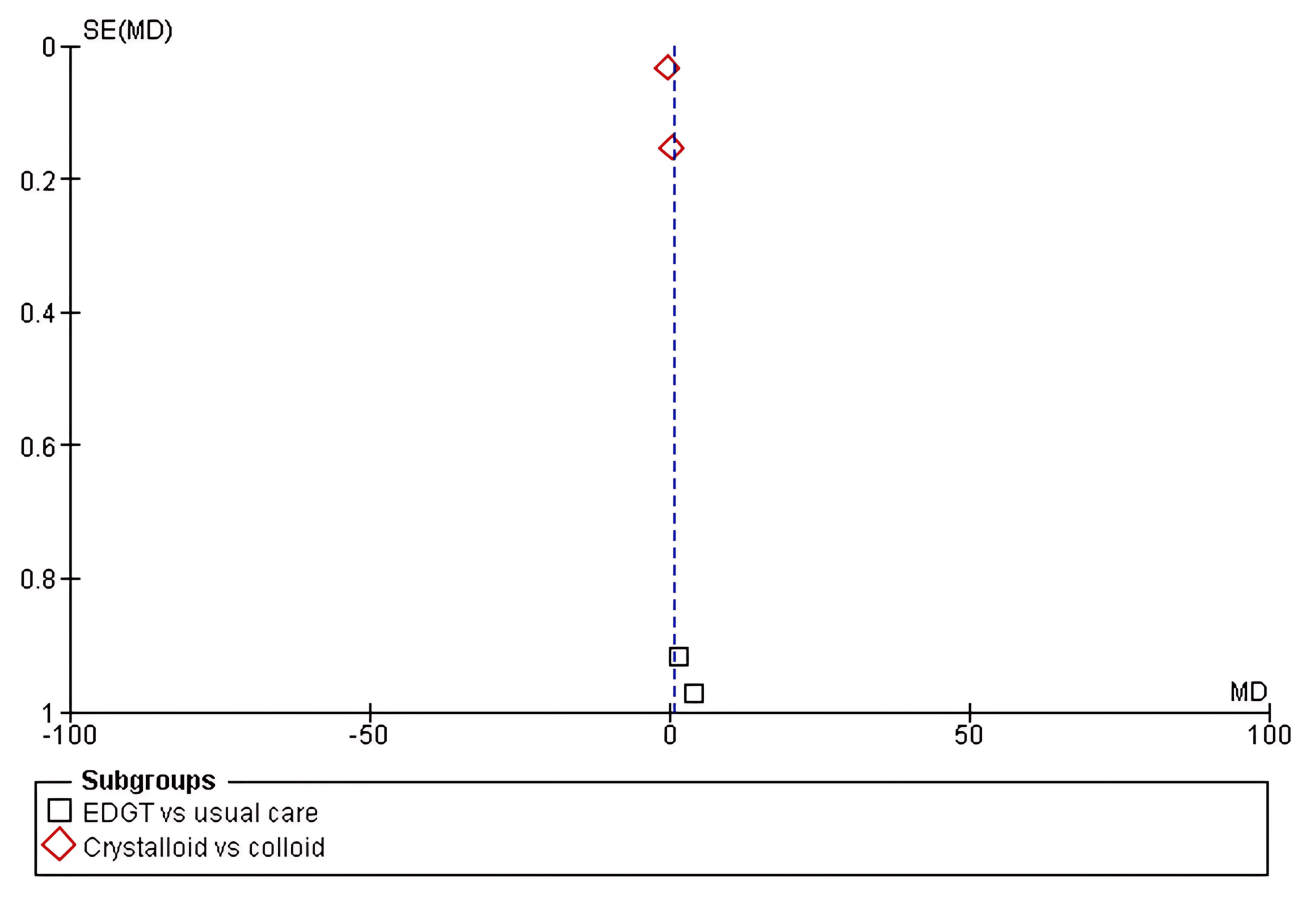
Funnel plot assessing publication bias across subgroups The x-axis represents the MD, and the y-axis shows the SE of MD. Squares indicate EGDT vs. usual care; diamonds indicate crystalloids vs. colloids. Symmetry suggests low publication bias. MD: mean difference; SE(MD): standard error of mean difference

Four of the eight included studies reported the length of ICU stay as an outcome. The pooled analysis showed a longer ICU stay in the first subgroup, EGDT versus usual care (MD 2.81, 95% CI 0.21-5.41; p = 0.05). In the second subgroup, crystalloids versus colloids, no difference was found (MD -0.02, 95% CI -0.61 to 0.57; p = 0.00001), with substantial heterogeneity (I² = 92%), as shown in Figure 8 and Figure 9.

Discussion

Fluid resuscitation strategies for critically ill patients may influence whether AKI occurs and how it can be treated. For fluid resuscitation, balanced crystalloids such as Plasma-Lyte appear to be the most beneficial, whereas the use of high-molecular-weight HES increases the incidence and risk of AKI and RRT [28,31]. Although fluid resuscitation remains an important strategy for preventing and treating AKI, its safety and benefits have been questioned. Treatment of septic shock with goal-directed therapy has not shown significant benefits in preventing AKI development or avoiding fluid overload [32]. Many studies have also suggested that fluid overload, especially during AKI, is associated with increased mortality [33,34]. Liberal fluid administration has been shown to worsen pulmonary function without providing renal protection. These findings emphasize the importance of prudent fluid management in critically ill patients to reduce the risk of AKI.

For critically ill patients, fluid management strategies should focus on carefully assessing individual responsiveness to fluids rather than solely on the type of fluid used. The response of each patient affects the outcomes of fluid management strategies such as EGDT and restrictive fluid therapy [35]. Fluid overload is a common consequence of aggressive resuscitation and can lead to prolonged hospital stays, organ dysfunction, and increased mortality [36]. Dynamic indicators of fluid responsiveness, such as stroke volume variation, may provide more accurate guidance for adjusting fluid delivery to patient needs [37]. This highlights the importance of continuously reassessing the patient’s status during resuscitation and adjusting therapy accordingly, rather than strictly adhering to fixed protocols. Identifying early signs of fluid overload and adjusting fluid administration may improve outcomes, underscoring the value of personalized management over standardized approaches [38]. Outcomes of different fluid types may also vary depending on patient-specific factors such as disease, gender, and age. For example, sepsis patients may be more susceptible to acidosis, which could increase mortality and AKI risk [39]. Although this meta-analysis included patients from various regions, additional subgroup or sensitivity analyses could not be performed due to the small number of studies [40]. Therefore, further research is needed to assess the effects of various fluids in different patient populations and disease conditions. In the meantime, individualized fluid management remains essential for every critically ill patient.

Fluid overload is common in critically ill patients and has been strongly associated with increased mortality and RRT use. Some studies suggest that normal saline may contribute to fluid overload due to its high sodium content [41,42]. While fluid amounts varied considerably among studies, it remains debated whether a positive or negative fluid balance worsens outcomes [43,44]. Thus, both fluid type and responsiveness should be considered in resuscitation strategies.

Previous studies have reported that, compared with normal saline, balanced crystalloids reduced mortality, AKI risk, and RRT use among critically ill patients receiving crystalloid therapy [31]. Zhang et al. [43] found that fluid overload increases mortality risk in AKI patients, while a meta-analysis by Semler and Rice [45] reported that using balanced crystalloids instead of saline did not significantly change mortality or kidney injury outcomes, suggesting that crystalloid choice may not be a decisive factor. These findings are consistent with the systematic review by Zampieri et al. [46], which found that excessive fluid administration in patients with renal impairment worsens outcomes, highlighting the importance of careful fluid management to improve prognosis.

Limitations

This study has several limitations. First, the number of studies comparing two fluid resuscitation strategies for preventing AKI was limited. More RCTs are needed for robust comparative evaluations. Although heterogeneity among subgroup analyses was low, this suggests limited rigor in the meta-analysis. Additionally, the low heterogeneity made it infeasible to conduct sensitivity analyses. Larger RCTs are required to provide stronger evidence on the comparative effectiveness of different fluid resuscitation strategies in preventing AKI.

## Conclusions

This meta-analysis found no significant difference in the incidence of AKI, mortality, or RRT requirement between crystalloids and colloids. Similarly, outcomes for EGDT versus standard care were only marginally different. The correlation between AKI incidence and either EGDT or crystalloids was weak. ICU length of stay was similar between crystalloid and colloid groups, whereas patients in the EGDT group stayed longer than those receiving standard care. Further studies are needed to confirm these findings and refine fluid management protocols in critical care.

## Figures and Tables

**Figure 10:**
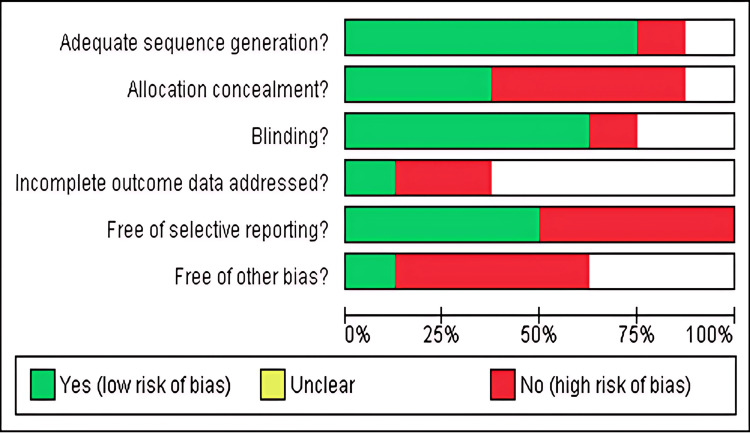
Representation of risk of bias graph

**Figure 11:**
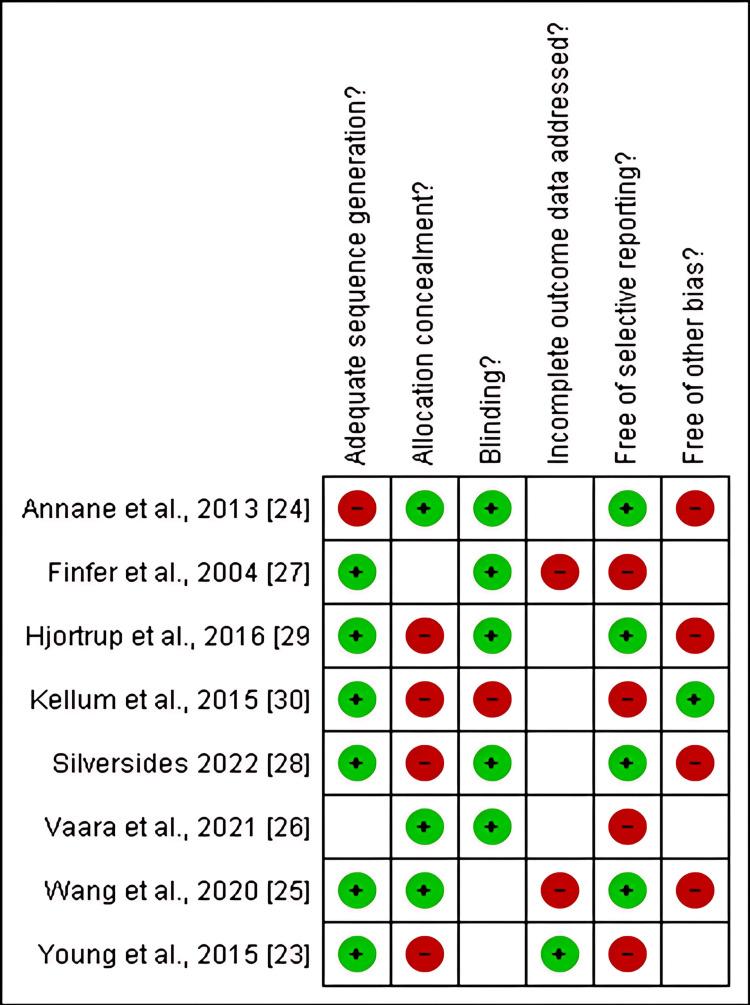
Representation of risk of bias summary

## Appendices

Appendix A 

Appendix B
